# Paclitaxel, Epirubicin and Capecitabine (TEX) as First-Line Treatment for Metastatic Breast Cancer: a Pilot Phase I/II Feasibility Study

**DOI:** 10.4137/cmo.s1027

**Published:** 2008-09-25

**Authors:** Z. Einbeigi, D. Bergström, T. Hatschek, M. Malmberg

**Affiliations:** 1 Department of Oncology, Sahlgrenska University Hospital, Göteborg, Sweden; 2 Roche AB, Stockholm, Sweden; 3 Radiumhemmet, Karolinska University Hospital, Stockholm, Sweden; 4 Department of Oncology, Helsingborg Hospital, Helsingborg, Sweden

**Keywords:** epirubicin, paclitaxel, capecitabine, first-line chemotherapy, metastatic breast cancer, phase I/II

## Abstract

Thirteen patients with untreated metastatic breast cancer received epirubicin 60 mg/m^2^, paclitaxel 155 mg/m^2^ (both day 1) and capecitabine 665 mg/m^2^ twice daily (days 1–14) every 21 days, with intra-patient dose escalation/reduction. Grade 3/4 events were infrequent. Nine patients (69%) achieved an objective response. Median time to progression and overall survival were 6.6 and 23.5 months, respectively.

## Introduction

The majority of patients with metastatic breast cancer (MBC) receive front-line chemotherapy. Anthracycline- and taxane-containing regimens are among the most active in MBC that is rapidly progressing or unresponsive to hormonal therapy (Biganzoli et al. 2002; Jassem et al. 2001; Nabholtz et al. 2003; Bonneterre et al. 2004; Sledge et al. 2003). Capecitabine demonstrates consistently high single-agent activity in pretreated MBC (Blum et al. 1999; Blum et al. 2001; Fumoleau et al. 2004; Reichardt et al. 2003) and is a promising first-line therapy (O’Shaughnessy et al. 2001; Stockler et al. 2007). In a randomized, phase III trial, first-line capecitabine significantly improved overall survival compared with ‘classical’ cyclophosphamide, methotrexate and 5-fluorouracil (CMF) (Stockler et al. 2007). Capecitabine has a favorable tolerability profile (Blum et al. 1999; Blum et al. 2001; Fumoleau et al. 2004; Reichardt et al. 2003; O’Shaughnessy et al. 2001; Stockler et al. 2007), including minimal myelosuppression, making it an appealing combination partner for other cytotoxics. Furthermore, capecitabine shows preclinical synergy with taxanes (Sawada et al. 1998). This observation has been substantiated by findings from a large, randomized clinical trial, which showed that capecitabine plus docetaxel significantly improved overall survival, time to disease progression and response rate compared with docetaxel alone in anthracycline-pretreated MBC (O’Shaughnessy et al. 2002). The combination of capecitabine and paclitaxel has demonstrated promising activity in phase II and III studies (Batista et al. 2004; Gradishar et al. 2004; Blum et al. 2006; Soto et al. 2006; Lück et al. 2007).

As current evidence suggests that three of the most active cytotoxic agents in MBC are the anthracyclines, taxanes and capecitabine, there is a rationale for studying the triplet combination of paclitaxel, epirubicin and capecitabine (TEX).

## Patients and Methods

This non-comparative, open-label study was designed to determine the optimal doses of each component drug and evaluate the safety and feasibility of TEX as first-line treatment for MBC. The study was conducted with ethics committee approval.

### Patient selection

Women >18 years old with documented MBC who had not received previous chemotherapy for MBC were eligible. Previous adjuvant chemotherapy was permitted if completed at least 12 months before relapse. Patients were to have at least one measurable lesion (at least one dimension ≥20 mm or ≥10 mm by conventional or spiral computed tomography [CT] scan, respectively); Eastern Cooperative Oncology Group (ECOG) performance score of 0–2; white blood cell (WBC) count ≥3.0 × 10^9^/l; platelet count ≥100 × 10^9^/l; serum creatinine ≤1.25 × upper normal limit (UNL); total bilirubin ≤1.25 × UNL; normal cardiac function; and a life expectancy of ≥3 months. All patients provided written informed consent.

Exclusion criteria included a history of neoplasm other than breast carcinoma, except for non-melanoma skin cancer or curatively treated carcinoma *in situ* of the cervix; pregnancy or lactation; known brain metastases; a history of cardiac arrhythmias and/or congestive heart failure or myocardial infarction; pre-existing motor or sensory neuropathy; severe hepatic impairment; severe renal impairment (creatinine clearance <30 ml/min); history of dihydropyrimidine dehydrogenase (DPD) deficiency; active infection; or other serious underlying medical condition.

### Drug administration

Study treatment consisted of combination therapy with paclitaxel, epirubicin and capecitabine. Epirubicin was given as a 30-min intravenous infusion on day 1 followed 30 min later by a 3-h intravenous infusion of paclitaxel; capecitabine was given orally twice daily for 14 days. Treatment was repeated every 3 weeks and continued until disease progression or unacceptable toxicity, or for as long as deemed appropriate by the investigator. All patients received premedication consisting of cetirizine 10 mg orally, 20 mg intravenous betamethasone. and 50 mg intravenous ranitidine.

### Dose adjustments

Starting doses in all patients were paclitaxel 155 mg/m^2^, epirubicin 60 mg/m^2^ and capecitabine 665 mg/m^2^ twice daily (level 0). Doses were escalated and reduced according to the tolerability of the treatment in each patient.

In the event of severe toxicity, doses were reduced to the preceding dose level according to Table 1, or if at dose level 0, at the discretion of the treating physician. Dose reductions of one or more agents were applied in the event of adverse events as follows: grade ≥3 hematological (epirubicin, paclitaxel); grade ≥2 mucositis or cardiac symptoms (epirubicin, capecitabine); grade ≥3 peripheral neuropathy, hypersensitivity reaction or arthralgia/myalgia (paclitaxel); grade ≥3 vomiting (epirubicin); grade ≥2 hand-foot syndrome or diarrhea (capecitabine). For all other treatment-related adverse events of grade ≥2, doses of the suspected drug(s) were reduced according to Table 1. Doses were escalated stepwise in the absence of toxicity, and maintained at the same dose if patients experienced grade 1 toxicity.

### Pretreatment assessment and follow-up

At baseline, patient history was recorded and cardiac investigations were performed (including electrocardiogram, and echocardiography/multigated radionuclide angiography [MUGA] scan if patients had signs or symptoms of cardiac disease at study entry). Physical examination, ECOG performance status and biochemistry tests (serum creatinine, total bilirubin, alkaline phosphatase, aspartate aminotransferase) were performed at baseline and on day 1 of each treatment cycle. Routine laboratory tests (hemoglobin, WBC and platelet count) were performed at baseline and on day 10, day 12 or 13 and day 15 of the first cycle, and at day 1 and the day of nadir for subsequent cycles.

Patients were screened by CT scan and bone scan and with magnetic resonance imaging (MRI) and bone X-ray if indicated. CT scan or MRI was performed at baseline, after every third cycle and at termination of study treatment. Patients underwent regular follow-up after discontinuing treatment until disease progression or death. Tumor response was evaluated according to Response Evaluation Criteria In Solid Tumors (RECIST) (Therasse et al. 2000), except for patients with bone metastases only, in whom tumor response was evaluated according to World Health Organization (WHO) criteria (WHO, 1979). Since the study was designed to assess feasibility rather than efficacy, this variation in response assessment was considered necessary and acceptable. Response duration was measured according to RECIST/WHO criteria (Therasse et al. 2000; WHO, 1979).

Toxicities were graded after each treatment cycle using National Cancer Institute Common Toxicity Criteria (NCI-CTC), version 2.0.

## Results

### Patient characteristics and treatment exposure

Thirteen patients were enrolled from 3 centers (Table 2). A total of 100 cycles of TEX were given (median 6 per patient, range 1–15). Reasons for discontinuing therapy were toxicity (n = 4), disease progression (n = 2), patient request (n = 5; 4 of whom were told at the start of treatment that they could elect to switch to ‘standard’ treatment after 6 cycles), complete remission (n = 1), or death from disease progression (n = 1). The patient with complete remission achieved this response after 3 cycles. The patient was switched to endocrine therapy after 4 cycles based on modest toxicity and the complete tumor response.

**Figure 1:**
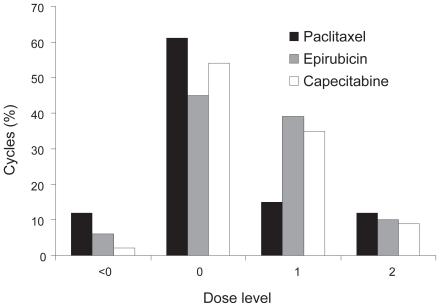
Proportion of cycles administered by drug and dose level.

Dose escalation was implemented in 10 patients, with epirubicin and capecitabine being most frequently escalated by one dose step. Figure 1 shows the number of cycles given at each dose level for each agent. Treatment with capecitabine alone or in combination with either paclitaxel or epirubicin was continued in 4 patients following termination of the triplet combination. The most commonly administered dose level for each drug was level 0, and therefore this dose level is recommended for further evaluation.

### Safety

**Figure 2:**
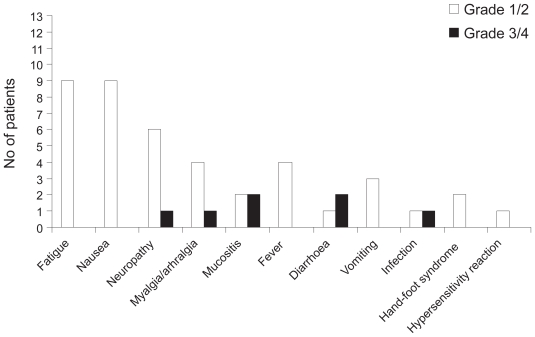
Non-hematological adverse events reported with paclitaxel, epirubicin and capecitabine (TEX) combination therapy.

The majority (86%) of adverse events were grade 1 or 2. The most commonly reported adverse events were fatigue, nausea, neuropathy and myalgia/arthralgia (Fig. 2). Grade 3/4 events were mucositis, diarrhea, infection, neuropathy and myalgia/arthralgia. Mean changes in WBC count are presented in Table 3. Seven patients experienced a total of 14 episodes of grade 3 (11 episodes) or 4 (3 episodes) leukopenia (5 patients experienced at least one grade 3 episode and 2 patients experienced at least one grade 4 episode). In all but one case, grade 3/4 leukopenia occurred at dose levels above level 0.

No patients required granulocyte colony stimulating factor support and none experienced cardiotoxicity, despite prior adjuvant anthracycline therapy in 3 patients.

### Efficacy

Nine patients achieved an objective response, including complete remission in a patient who received 4 cycles of TEX (paclitaxel and epirubicin at dose level 0, capecitabine at dose level 1). Two patients had stable disease. One showed disease progression, and 1 was not evaluable due to early toxicity. Median time to treatment failure was 4.9 months (range 0.4–11.8). At 19 months’ median follow-up, the median time to tumor progression and median overall survival were 6.6 (range 2.2–15.5) and 23.5 (range 7.3–65+) months, respectively.

## Discussion

In this pilot, phase I/II feasibility study, a regimen of paclitaxel 155 mg/m^2^, epirubicin 60 mg/m^2^ and capecitabine 665 mg/m^2^ twice daily was identified as a feasible, tolerable and active first-line treatment for MBC. Consistent with the known toxicities of each agent, the most frequent adverse events were fatigue, nausea, sensory neuropathy and myalgia/arthralgia. Most events were mild or moderate in intensity, with grade 3/4 toxicities comprising only 14% of reported events. In contrast to an early study of docetaxel-epirubicin-capecitabine (Venturini et al. 2003), myelosuppression was manageable in this study, probably due to the lower anthracycline and taxane doses. Only 7 patients experienced grade 3/4 leukopenia and none experienced febrile neutropenia, although infection was reported in a patient receiving the majority of courses of capecitabine at dose level 0 and paclitaxel and epirubicin at dose level 2.

Although this very small study was not designed to determine efficacy, we observed objective responses in 9 patients (69%). This is similar to the 67% response rate reported in a phase II study evaluating docetaxel-epirubicin-capecitabine in 33 women with MBC (Venturini et al. 2003). Recently reported results of a randomized, phase III trial comparing docetaxel-epirubicin-capecitabine with docetaxel-epirubicin showed response rates among patients with stage IV disease of 67% and 53%, respectively (Mansutti et al. 2008). Overall, our results are in accordance with those reported from other trials (Venturini et al. 2003; Mansutti et al. 2008), although the very small number of patients in our study means that such comparisons should be treated with caution. In addition, it is important to note that in our trial the capecitabine dose was considerably lower than the 1000 mg/m^2^ twice daily dose used in phase II and III trials evaluating docetaxel-epirubicin-capecitabine.

Currently, the most commonly used chemotherapeutic agents for the treatment of breast cancer are anthracyclines and taxanes. Capecitabine is highly active and may further improve outcomes, possibly through synergy between capecitabine and taxanes and with the benefit of non-overlapping toxicity. In chemo-naïve patients with good performance status, the primary aim is to achieve disease and symptom control. Consequently, TEX is being compared with paclitaxel-epirubicin as first-line therapy for MBC in an ongoing Swedish phase III trial.

Increasingly, drugs with defined targets (such as bevacizumab in combination with a taxane (Miller et al. 2007; Miles et al. 2008)) are becoming an important part of breast cancer treatment. With further study, it should become clear how these agents should be integrated into standard first-line therapy for MBC.

## Figures and Tables

**Table 1:** Dose levels for paclitaxel, epirubicin and capecitabine.

Dose level	Paclitaxel (mg/m^2^, d1) 3-h infusion	Epirubicin (mg/m^2^, d1) 30-min infusion	Capecitabine (mg/m^2^, b.i.d.) d1–14 p.o.
0 (starting dose level)	155	60	665
1	175	75	825
2	200	90	1000

b.i.d., twice daily; p.o., orally.

**Table 2:** Baseline patient characteristics (n **=** 13).

Characteristic	
Median age, years (range)	50 (43–65)
ECOG performance status, n (%)	
0	5 (38%)
1	8 (62%)
Hormone receptor status, n (%)	
ER and/or PgR positive	10 (77%)
ER and PgR negative	2 (15%)
Unknown	1 (8%)
Histology, n (%)	
Ductal	12 (92%)
Lobular	1 (8%)
Median no. of metastatic sites (range)	2 (1–3)
Metastatic sites, n (%)	
Bone	7 (54%)
Liver	5 (38%)
Lung	3 (23%)
Previous therapy, n (%)	
Radiotherapy	11 (85%)
Hormonal therapy	8^a^ (62%)
Adjuvant chemotherapy	5^b^ (38%)

a Adjuvant therapy (n = 2); first-line therapy (n = 6).

b Tailored 5-fluorouracil, epirubicin and cyclophosphamide (n = 2), cyclophosphamide, methotrexate and 5-fluorouracil (n = 2) and doxorubicin and docetaxel (n = 1).

ECOG, eastern cooperative oncology group; ER, estrogen receptor; PgR, progesterone receptor.

**Table 3:** White blood cell count at baseline, prior to next cycle and at nadir (n **=** 13).

	Mean	95% confidence interval (range)
Baseline	7.73	6.18–9.28
Before next cycle	5.30	5.00–5.60
Nadir	2.67	2.51–2.83
